# Physical predictors for retention and dismissal of professional soccer head coaches: an analysis of locomotor variables using logistic regression pipeline

**DOI:** 10.3389/fspor.2023.1301845

**Published:** 2023-11-20

**Authors:** Honorato Sousa, Rabiu Muazu Musa, Filipe Manuel Clemente, Hugo Sarmento, Élvio R. Gouveia

**Affiliations:** ^1^ Research Unit for Sport and Physical Activity, Faculty of Sport Sciences and Physical Education, University of Coimbra, Coimbra, Portugal; ^2^ Centre for Fundamental and Continuing Education, Universiti Malaysia Terengganu, Kuala Nerus, Malaysia; ^3^ Escola Superior Desporto e Lazer, Instituto Politécnico de Viana do Castelo, Rua Escola Industrial e Comercial de Nun’Álvares, Viana do Castelo, Portugal; ^4^ Gdansk University of Physical Education and Sport, Gdańsk, Poland; ^5^ Sport Physical Activity and Health Research & Innovation Center SPRINT, Melgaço, Portugal; ^6^ Department of Physical Education and Sport, University of Madeira, Funchal, Portugal; ^7^ LARSyS, Interactive Technologies Institute, Funchal, Portugal

**Keywords:** replacement, head coaches, external load, locomotor variables, performance

## Abstract

**Introduction:**

Soccer has enormous global popularity, increasing pressure on clubs to optimize performance. In failure, the tendency is to replace the Head coach (HC). This study aimed to check the physical effects of mid-season replacements of HCs, investigating which external load variables can predict retention or dismissal.

**Methods:**

The data was collected in training and matches of a professional adult male soccer team during three complete seasons (2020/21-2022/2023). The sample included 6 different HCs (48.8 ± 7.4 years of age; 11.2 ± 3.9 years as a HC). The 4 weeks and 4 games before and after the replacement of HCs were analysed. External load variables were collected with Global Positioning System (GPS) devices. A logistic regression (LR) model was developed to classify the HCs' retention or dismissal. A sensitivity analysis was also conducted to determine the specific locomotive variables that could predict the likelihood of HC retention or dismissal.

**Results:**

In competition, locomotor performance was better under the dismissed HCs, whereas the new HC had better values during training. The LR model demonstrated a good prediction accuracy of 80% with a recall and precision of 85% and 78%, respectively, amongst other model performance indicators. Meters per minute in games was the only significant variable that could serve as a potential physical marker to signal performance decline and predict the potential dismissal of an HC with an odd ratio of 32.4%.

**Discussion:**

An in-depth analysis and further studies are needed to understand other factors' effects on HC replacement or retention.

## 1. Introduction

Success in professional soccer heavily depends on winning games and achieving favourable results, which can create significant pressure for players and head coaches. As a result, head coaches are predominantly evaluated by the match results. In elite soccer, it is common practice to dismiss the head coach after an extended period of negative results or poor performance outcomes, unfulfilled expectations, dissatisfaction of stakeholders and fans, and attempting to change the adverse streak (1–4).

Commonly, club management promotes the change with the aim to increase collective performance and sub-standard position in the league table, stimulate a motivational growth of players in pre- and post-game contexts, and calm down the dissatisfaction of stakeholders, sponsors, and fans (1, 5, 6).

Despite the significant uncertainty it introduces into organizational development, financial stability, and, most critically, the development of players and teams, elite club management still chooses to replace head coaches mid-season with limited data supporting their decision-making (7). Currently, there are available data that indicate that the head coach turnover rate in professional soccer is very high (4, 8, 9). During the past 10 seasons, in the top five professional European men's soccer leagues (English Premier League, Spanish La Liga, German 1st Bundesliga, Italian Serie A, and French Ligue), in-season head-coach changes occurred frequently, registering 548 in-season head coach replacements (4).

Although the topic of head coach turnovers has been studied for many years, it is still uncertain whether the change of coach affects teams' performance, and if so, how and in what way (10, 11). The available research that analyses the effect of changing coaches has focused mainly on the effects on teams' sports results (4, 12, 13), and the scientific evidence is limited concerning studies that analyse the effects of changing coaches on the locomotor responses of players and physical training and match performance (14, 15). The existing results revealed that the change could potentially affect the sporting and physical performance of a professional soccer team, but with a limited time span, in some cases, even worsening the teams' performance (14, 16).

The increased congestion of the competitive sports calendar caused an escalation in the loads on elite athletes. As a result, training monitoring has emerged as a vital process for evaluating individual player responses and tailoring individual and group training regimens in elite team sports (17–19). Advancements in sports technology, coupled with the accessibility of cost-effective solutions, have paved the way for the widespread adoption of player monitoring systems within professional clubs. These systems are designed to precisely measure and qualify the extent to which players are subjected to physical stresses during training sessions and matches (20–22).

The training and match loads can be split up into external (physical) and internal (psychophysiological) loads, as they can allow controlling training and game session demands (23). The external load is defined as the total amount of locomotor and mechanical work performed during training sessions or matches, being monitored (for example) by Global Positioning Systems (GPS), commonly measuring performance variables such as distances, speeds, and accelerations/decelerations (among others). It has been more appropriate in the planning and prescribing of the training process (17, 18).

High-performance soccer head coaches' work occurs in a dynamic and complex context, where they play a central role in the coach-athlete-performance process. Among the responsibilities of a head coach, ongoing monitoring, and manipulation of a player's physical loads within the context of a periodized preparation program by defining objectives within training sessions, microcycles, and game context is a fundamental task (7, 9, 24).

Given that changes in the coaching staff are a common occurrence in professional soccer, such personnel changes can impact the physical performance of players in training and matches, primarily because of variances in training philosophies. For example, the use of small-sided games, when compared with the use of exercises with large spaces, has revealed significant variations in external training loads. Furthermore, differences in training session structure and the predominant stimuli incorporated in developing diverse match strategies can lead to varying values in external load control metrics, both within and among coaches (25–29).

The importance of external load training measurements in the training process and match performance has been highlighted in the literature as a fundamental tool for head coaches to work (30–32). Even so, despite many factors (tactical, technical, or physiological, among others) affecting the players' performance, currently, winning is the most important aspect in high-level soccer, with performance being assessed through victory or defeat in high-level soccer (2), making this connection between external physical factors and retention/dismission rather speculative. Nevertheless, the physiological component of the external load, when trying to understand if there could be some type of pattern that could symbolize a physiological marker to function as a possible reference for a drop in performance, could be interesting to comprehend. In this sense, coaches and their technical teams, when noticing a drop in metrics, possibly in those that could be referenced as the most important physiological markers, can try to create strategies to reverse this situation. Therefore, this study aims to verify the physical effects of mid-season replacement of HCs and if the locomotive variables related to the external load and the physical performance of professional soccer players in training and competition can predict the retention or dismissal of professional soccer head coaches.

## 2. Material and methods

### 2.1. Study design and setting

This is a cross-sectional observational study. The assessments and data collection occurred in all training sessions and official matches of a professional adult male soccer Club during the span of three complete seasons (2020/21, 2021/22 and 2022/2023). To carry out the analyses on the possible variations and differences in performance, the 4 weeks and 4 games before the replacement of the old coach will be analysed, as well as the first 4 weeks and 4 games after the arrival of the new coach. The 4 weeks (about 20 training sessions) were determined based on the sample calculation (GPower 3.1), which indicates that 12 training sessions (effect size and P defined at 0.40 and 0.05, respectively) will allow to produce a statistical effect significant (power of 0.90). In this context, representation is determined by the frequency of training sessions and games rather than the number of players involved. It is crucial to grasp the innovative framework of this study, which places a strong emphasis on the comprehensive physiological processes. This approach aims to unravel the intricacies of the team's preparation for competition, achieved by considering the training sessions in conjunction with their corresponding week's games rather than fixating solely on the team's sporting outcomes, such as wins or losses.

### 2.2. Participants

During this three-season period, the team had a total of 6 different head coaches (48.8 ± 7.4 years of age; 11.2 ± 3.9 years as a head coach; all with UEFA Pro License). In the 1st season, there were a total of three head coaches; in the 2nd season, the head coach that finished the 1st season started, but another head coach finished the season, totalling 2 head coaches; in 3rd season, the head coach that ended the 2nd season, started but not ended, having the team had 2 two more head coaches until the end of the season. All the coaches were dismissed for reasons related to bad results and poor performances. The competitive record and other relevant performance indicators of this club's competitive path between the 2020/2021 and 2022/23 seasons are in the public domain. Data was collected from the sports platforms zerozero.pt and transfermarkt.com. The consistency of the information in both sources (date of signing and dismissal of coaches, matches, results) was identical, validating the data presented. All procedures applied were approved by the Ethics Committee of the Faculdade de Motricidade Humana N° 34/2021. The investigation was conducted following the Declaration of Helsinki.

### 2.3. Instruments

The external load variables were collected using two Global Positioning System (GPS) devices (1st season - 10-Hz GPS unit EVO, Catapult; 2nd and 3rd seasons – STATSports APEX, 10 Hz augmented GNSS). The GPS devices were put in a skin-tight bag in the thoracic region between the scapulae. Participants used the GPS device in training and match situations (only official and competitive games from a league with 34 fixtures).

### 2.4. Variables under study

In this study, we have variables of two different matrices: volume and intensity variables. In the volume variables, we have: (1) total distance in game (TD-G) and training (TD-T), which provides a good global representation of the volume of exercise (walking, running) and is also used to assess an individual's contribution relative to a team effort; (2) Total distance above 14 km/hr (zone 4) in game (DB4-G) and training (DB4-T), 18 km/hr (zone 5) in game (DB5-G) and training (DB5–6) and 24 km/h (zone 6) in game (DB6-G) and training (DB6-T), which account for the total distances covered at the different speeds indicated; (3) high speed running in game (HSR-G) and training (HSR-T), the sum of the distance covered when in speed zones 5 and 6 (above 18 km/hr and 24 km/hr); (4) accelerations in game (AC-G) and training (AC-T), the number of times that players acceleration is greater than the acceleration threshold (3 m/s/s) for at least 1 s; 5) decelerations in game (DC-G) and training (DC-T), the number of times that players deceleration is greater than the deceleration (3 m/s/s) threshold for at least 1 s.

Regarding the intensity variables, we have: (1) maximum speed in game (MS-G) and training (MS-T), which measures top speed as the maximum speed sustained for at least half a second; (2) meters per minute in-game (MM-G) and training (MM-T), which counts the total distance in meters covered, divided by the duration of the session/exercise.

Additionally, a training intensity control variable was introduced, specifically focusing on internal load, referred to as the rate of perceived exertion (RPE-T), validated using the scale developed by Foster et al. (33). Data collection for RPE-T was organized in a manner that allowed each player to provide their assessment individually, separate from the other team members. Immediately after the conclusion of a training session, players utilized a tablet device to indicate their personal perceived effort on a scale ranging from 1 (minimal) to 10 (maximal).

### 2.5. Data treatment and statistical analysis

The Shapiro–Wilks normality test was performed, verifying the normal distribution of the data (*p *> 0.05). The data on the locomotive performance of the team for 3 consecutive seasons was gathered and pre-processed. The data were also normalized to avoid bias from different units of measurement. The data included forty games and training sessions per week for each season. The average locomotive performances of all the players on a game-by-game and training-by-training basis were extracted for the statistical analysis. We assigned the coaching job status (retained, dismissed) based on whether they were the last four or the first four games of the coach. We considered the last four games as the dismissal period and the first four games as the retention period. Consequently, we aim to predict the likelihood of the coach's dismissal or retention concerning the team's training and game locomotive performances.

### 2.6. Development of logistic regression model pipeline

A logistic regression model (LR) was employed to ascertain the efficacy of the models in classifying the statuses of coaches, i.e., dismissed or retained. A five-fold cross-validation technique was used in Field (34) to validate the model's performance. This technique is considered suitable as it is shown to be useful in mitigating overfitting problems, especially when there is relatively minimum observation in the dataset. The average performance over all the folds is then computed. The data was split into a ratio of 70:30 for training and test sets (35, 36). Twenty-seven sets of data were used to train the model, whilst the remaining 13 observations were used for testing to evaluate the predictability of the classifiers in determining dismissed or retained. The cutoff point for the machine learning model parameters is set at 60%. This aligns with previous research showing that a model with an accuracy of 60% or higher is considered good and acceptable in social science-related research (37). The Pycaret libraries were evoked to develop the LR model via Spyder IDE. Other statistical analyses were implemented using the add-in software and Orange Canvas version 3.4.0 and XL STAT add-in software version 2014 for Windows.

#### 2.6.1. Model evaluation

In this study, we evaluated the logistic regression (LR) model using several performance measures: classification accuracy (ACC), an area under the curve (AUC), recall, precision (PREC), F1 score, Kappa, and Matthew's correlation coefficient (MCC). ACC is the fraction of correctly classified instances. AUC is a curve that shows the model's ability to separate classes. A recall is the proportion of true positives among actual positives, while PREC is the proportion of true positives among predicted positives. The F1 score is the harmonic mean of PREC and recall and measures the average accuracy for both classes. Kappa measures how well the classifier matches the ground truth data, adjusting for random accuracy. MCC is a discrete version of Pearson's correlation coefficient that ranges from 1 to 1 and measures the quality of binary classification. The confusion matrix shows the correctly and incorrectly classified instances between the classes. Moreover, we conducted a sensitivity analysis using significance attribute evaluation to examine the training and game locomotive variables that could best contribute to the model accuracy. A multivariable binary logistic regression was applied using the output from the significance evaluation to identify the significant locomotive variables that could predict the coaches' dismissal or retention by means of odd ratio analysis. We used these performance measures to evaluate the LR model's performance in solving the classification problems in this study.

## 3. Results

Table 1 tabulates the descriptive statistics of the investigated parameters. The mean and standard deviation of the locomotive variables are presented. The coaches have each managed 20 games (20 games assigned to both the dismissed and the retained coaches). It is noteworthy that the game results vary among the coaches. The retained coaches have recorded 8 wins, 5 draws and 7 defeats, while the dismissed coaches had a record of 1 win, 5 draws and 14 defeats.

**Table 1:** Descriptive statistics of the study variables.

Variables	Units of Measurement	Coach Status Groups	
		Mean ± (SD)	
		Dismissed (*n* = 20)	Retained (*n* = 20)
AC-T	Frequency: *n*	35.55 (10.29)	37.10 (14.85)
DC-T	Frequency: *n*	35.15 (7.32)	36.80 (11.95)
TD-T	Meters	4,323.30 (547.97)	4,213.05 (656.84)
MM-T	Meters	69.70 (9.48)	69.50 (7.76)
DB4-T	Meters	416.25 (68.32)	427.20 (104.46)
DB5-T	Meters	238.00 (77.38)	273.85 (79.47)
DB6-T	Meters	38.25 (13.74)	43.75 (18.29)
HSR-T	Meters	276.30 (88.30)	311.40 (87.36)
MS-T	Km/h	26.70 (0.98)	26.70 (1.49)
RPE-T	Reporting scale 1–10	4.45 (0.69)	4.55 (0.69)
AC-G	Frequency: *n*	43.20 (15.21)	46.85 (20.06)
DC-G	Frequency: *n*	56.40 (11.30)	56.90 (15.47)
TD-G	Meters	7,613.95 (956.22)	7,359.90 (1,206.84)
MM-G	Meters	92.85 (7.45)	86.35 (7.52)
DB4-G	Meters	986.75 (116.53)	928.05 (205.32)
DB5-G	Meters	602.35 (81.22)	579.10 (117.46)
DB6-G	Meters	140.35 (25.81)	149.45 (49.43)
HSR-G	Meters	733.25 (111.58)	728.45 (161.16)
MS-G	Km/h	30.10 (1.07)	29.95 (0.83)

Table 2 summarizes the performance of a logistic regression model in predicting the dismissal or retention of coaches. The predictive model achieved a mean accuracy score of 80%, indicating a good prediction of the coaches' statuses. The Area Under the Curve (AUC) was 0.90, which signifies perfect modelling in predicting coaches' statuses. The F1 or F Score is a weighted average score that correlates between Precision and Recall. The table reports that the Precision and Recall scores were 0.85 and 0.78, respectively. These scores demonstrate that the model predicted more than 85% of positive cases and correctly identified 78% of the positive classes. The *κ* score obtained by the model was 0.68, which indicates reasonable reliability, while Matthew's Correlation Coefficient (MCC) demonstrated a good prediction of 0.70. Overall, these findings suggest that the logistic regression model performed well in predicting the dismissal or retention of the coaches with respect to the training and games locomotive performance variables.

**Table 2:** Performance evaluation of the logistic regression model for predicting the status of coaches from game and training locomotive variables.

	Accuracy	AUC	Recall	Prec.	F1	*κ*	MCC
Mean	0.8000	0.9000	0.8500	0.7833	0.7833	0.6800	0.7000
Std	0.2667	0.3000	0.3202	0.3500	0.3167	0.4118	0.4000

**Figure 1:**
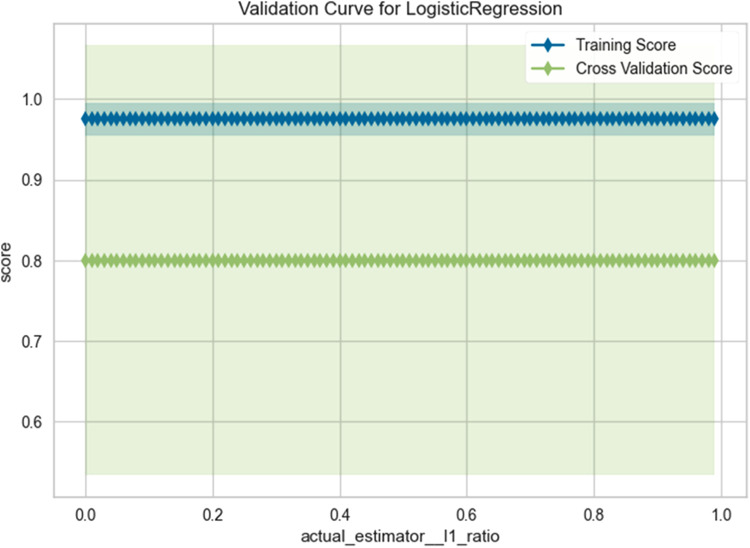
Training score of the model is at 98% while cross-validation stood at 80%.

Figure 1 shows the training and cross-validation scores of the model on the training dataset. It can be seen from the figure that the training score was higher than the score of cross-validation at 0.98 and 0.80, respectively. The drop in scores after cross-validation can be explained by data splitting for training and testing. It could be speculated that the training score before cross-validation implementation might have included noises and errors, which led to relatively high accuracy (38). However, the model was adjusted during the cross-validation stage, where the prediction accuracy score yielded 80%, indicating the efficiency and best fit of the formulated model.

**Figure 2:**
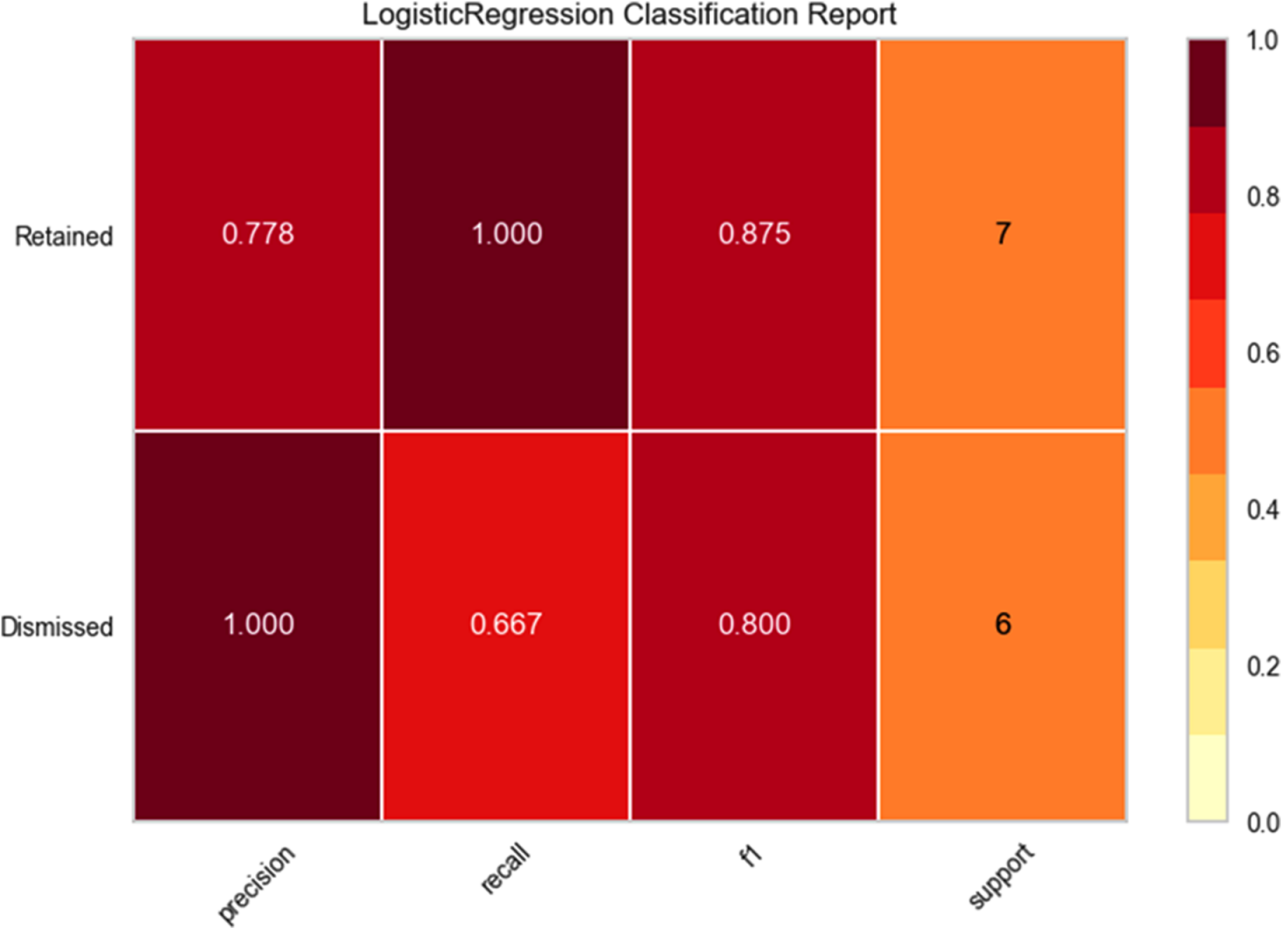
Precision, recall and F1 scores of the model for the classification task: Retained coaches (Precision = 0.78, recall = 1, F1 = 0.88); Dismissed coaches (Precision =1, recall = 0.67, F1 = 0.80).

Figure 2 highlights the logistic regression analysis results for classifying coaches' statuses after cross-validation. It is important to note that cross-validation is a process of validating the effectiveness of the formulated model on untrained data. In this stage, the new sample of untrained data was tested using the previously formulated logistic regression model to examine the developed model's accuracy. The F1, precision and Recall scores reduce as cross-validation occurs. For retained, the precision is shown to be 0.78, with a recall of 1.0 and F1 score of 0.88, while for the dismissed, a precision of 1, recall of 0.67 and F1 score of 0.80 were observed.

**Figure 3:**
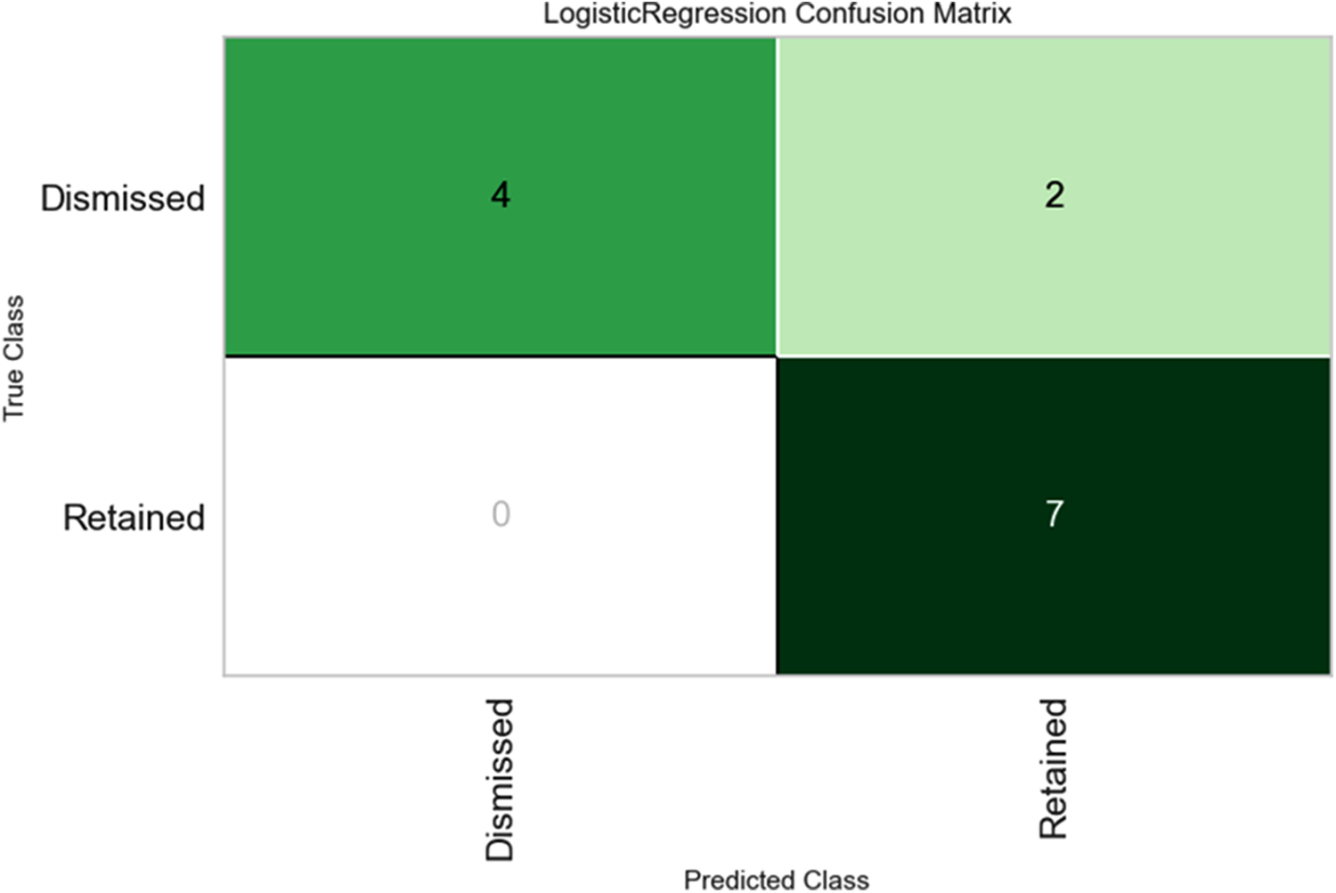
Retained coaches all 7 correctly classified; Dismissed coaches 4 correctly classified and 2 misclassified.

Figure 3 shows the confusion matrix of the developed model after cross-validation was performed. This technique was employed to evaluate the classifier's performance in predicting the dismissal or retention of the coaches using the reserved on unseen data. The model has correctly predicted 4 out of 6 dismissed, indicating 2 misclassifications. Interestingly, the model correctly predicted all 7 retained coaches without misclassification. Overall, it is apparent that the model performed well in the classification task against the test data despite a relatively low number of observations.

### 3.1. Sensitivity analysis: identification of most significant training and games locomotive variables towards the model accuracy

**Figure 4:**
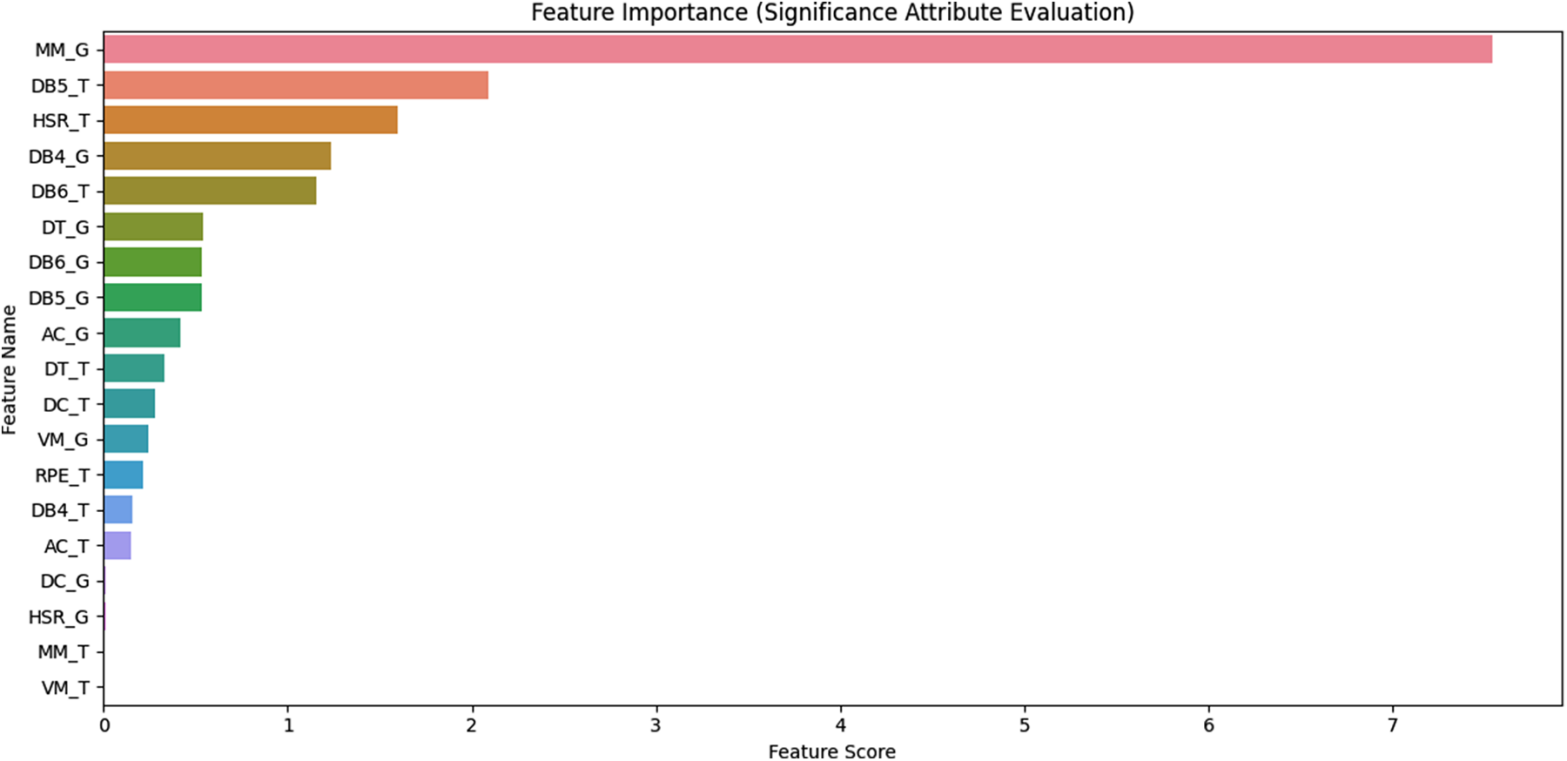
A total of 10 locomotive variables identified as highly contributing towards the model performance.

Figure 4 demonstrates the graphical visualization of the variable's contribution towards the performance of the model pipeline via the feature importance plots. The feature important plot analysis was used to determine the features or locomotive variables that contributed significantly towards the model performance. From the figure, it can be observed that 10 out of the 19 locomotive variables contributed higher towards the model performance (>0.5). Hence demonstrating the importance of the variables towards the dismissal or retention of the coaches. These variables were further analysed using multivariate logistic regression analysis to determine their contribution to the probability of dismissal or retention of the coach based on odd analysis.

Table 3 shows the multivariable binary logistic regression model performed to ascertain the significant disciplinary measures that could explain the statuses of the coaches. In this analysis, all locomotive games and training variables identified via the features important plots were used. The overall model presented a well-fitting value (Hosmer-Lemeshow >.05), a good classification accuracy (83%), and its discriminant capacity was also notable, with an AUC of 91% at a 95% confidence level. The model accounts for 63% odds for the overall events occurrences, i.e., dismissal or retention based on the games and training locomotive variables examined (Negelkerke *R*^2^ = 63.00).

**Table 3:** A multivariate LR sensitivity analysis of significant locomotive variables determining dismissal or retention of coaches.

						95% Confidence Interval	
Predictors	B	SE	Z	p	Odds ratio	Lower	Upper
Intercept	40.657	17.574	2.313	0.021	4.541	499.088	4.132
DT_G	−0.002	0.002	−0.909	0.363	0.998	0.995	1.002
MM_G	−0.392	0.181	−2.165	0.003*	0.676	0.474	0.964
DB4_G	0.007	0.010	0.76	0.447	1.007	0.989	1.026
DB5_G	−0.011	0.014	−0.825	0.409	0.989	0.963	1.016
DB6_G	0.063	0.038	1.649	0.099	1.065	0.988	1.147
AC_G	0.082	0.082	1.011	0.312	1.086	0.926	1.274
DT_T	−0.002	0.001	−1.462	0.144	0.998	0.995	1.001
DB5_T	0.029	0.215	0.136	0.892	1.03	0.676	1.568
DB6_T	0.042	0.222	0.19	0.849	1.043	0.675	1.611
HSR_T	−0.023	0.214	−0.109	0.913	0.977	0.642	1.487

* *p* < 0.05; Nagelkerke *R*^2 ^= 0.63; Hosmer Lemeshow (*p *= 0.98); AC = 83%; AUC = 0.91.

We found that only one locomotive variable significantly explained the coaches' status (*p* < .05): MM-G, which could predict the coaches' dismissal or retention based on the *p*-values and odd ratio analysis. In essence, for a one-unit decrease in meters per minute of a player during a game, the likelihood of the coach's dismissal increases by 32.4% [OR = 0.676, 95% CI (0.474–0.964)]. The results further revealed that the other 8 locomotive variables do not have significant effects on the odds of the dismissal or retention of the coaches (*p* > 0.05) (39).

## 4. Discussion

The effects of the HC replacement on the locomotor component are diverse and require careful analysis. There are two different contexts under consideration: the physical performances of the players in training and competition. In competition, the players performing under the commands of the dismissed HCs showed higher values in 6 of the 9 variables of the external load under analysis (MM-G; TD-G; DB4-G; DB5-G; MS-G; HSR-G). Under the guidance of the retained HC, the players demonstrated higher overall values in 3 out of the 9 variables (DB6-G; AC-G; DC-G).

In training, the players exhibited better performances under the responsibility of the retained HCs, with higher values in 6 of the 9 external load variables under consideration (DB4-T; DB5-T; DB6-T; HSR-T; AC-T; DC-T). Meanwhile, when training under the commands of the dismissed HCs, players revealed greater values in 2 of the 9 variables (TD-T; MM-T). The MS-T variable had the same value for both statuses of HCs in training. About the only internal load variable under analysis, the RPE-T, the players reported higher values in the training sessions under the responsibility of the retained HCs.

Focusing on differentiating between intensity and volume metrics, it is possible to check that the dismissed coaches had higher values in 3 of the 4 variables of the intensity-related ones (MM-G; MM-T; MS-G), unrelatedly of the context of observation, while the remaining variable of intensity (MS-T), presented equal values, regardless of the status of the coach. In the volume-related ones, in training, it was possible to perceive that the retained coaches had higher values in all the variables except for 1 of them (TD-T). In competition, the retained coaches presented higher values in 3 variables related to volume (DB6-T; AC-G; DC-G), while the dismissed ones presented superior values in 4 variables (DT-G; DB4-G; DB5-G; HSR-G).

Concerning the single variable for internal load under analysis, the RPE-T, the training sessions under the guidance of the retained HCs showed a superior perception of effort reported by the players, but with very close values. Even so, the superior values can be explained by increased motivation in the substitute players to show their ability to the new coach or in the usual starters by trying to maintain the status within the team (16).

Guerrero-Calderón et al. (16) is one of the few studies that analysed the effects that a change of HC causes on the physical performance of professional soccer players. Considering both training and competition contexts in the context of mid-season HC replacement, this study suggests that such replacements did not improve players' physical performance, whether in training or during competitions. The study's main finding revealed that players could modify their physical performance behaviours after the arrival of a new HC compared with the values registered with the dismissal HC. Similar performance between HCs was found in the context of competition. On the other hand, in training sessions, players displayed greater high-intensity activity with the dismissal HC. This could be explained by the possible different types of tasks in the training methodologies proposed by the HCs (large playing areas or reduced spaces require different efforts) or even by the variations of the playing style (playing different formations like 3:5:2 or 4:3:3, among others), booth factors that are proven to influence the training responses of the players (40).

These results contrast the data presented in the study of Radzimiński et al. (15), whose purpose was to identify potential changes in match results and physical match performance before and after the HC replacement. Regarding the physical match performance, there were significant improvements in some external load variables (total distance, high-speed running, meters per minute and number of high-intensity runs) immediately noticed after the HC turnover. As mentioned above, this increase in physicality in-game action could be triggered by an enhanced motivation caused by the arrival of the new HC, with the substitute players trying to prove their value to the team. In contrast, the usual starters prove that they deserve to keep their condition (16). However, the significant finding was related to a short-term positive effect on the physical match performance, but this effect disappeared after the first 5–10 games of the new HC.

When comparing our results with these two studies, relating to the match performance effects of the HC replacement, it is possible to check that we had more variables of the external load with totals lower than those recorded prior to dismissal, in some way contradicting the results of Radzimiński et al. (15) study (especially when relating with total distance, high-speed running and meters per minute). However, some critical external load variables are strictly related to game intensity. Accelerations, decelerations, total distance above 24 km/h and player load registered higher values with the new HCs, proving to be an important aspect to consider when formulating conclusions for being more appropriate in the planning and prescription of the training process, as mentioned before (17, 18).

When analysing the training context, the findings appear somewhat comparable to those presented by Guerrero-Calderón et al. (16). Players exhibited greater high-intensity activity under the dismissed coaches during training. However, our findings diverged in a competition setting, where the dismissed coaches displayed higher high-intensity activity rather than both coach groups performing similarly.

The MM-G, covered individually by the players, was the only locomotive variable with significant meaning when trying to explain the possible scenarios for the coaches' status. For each one-unit decrease in MM-G, the probability of the coach's dismissal increases by 32.4%. These results show the importance that this metric, directed towards the game's intensity, presents in the panorama of modern soccer. Looking at the results presented for the same metric in training, we can identify a relatively large distance between the medium values obtained in the two realities under analysis. With a weekly average of 69.70 meters in training in the dismissed coaches and 69.50 meters in the retained coaches, there is a difference of 23 meters for the dismissed coaches and almost 17 meters for the retained coaches when comparing the values in game and training for each status. This can be easily explained by the regular managing of the load intensities throughout the week of preparation for the game because it is not recommended to train throughout the week at the same intensity every day, let alone at the intensity of a game near the game itself (41, 42).

As previously shown in another study (16), players have displayed greater high-intensity activity with the dismissal of HC, which can be explained by methodological and game strategy differences, as earlier mentioned. Nevertheless, it is plausible that the heightened intensity can be attributed to the HC's imperative to push for more vigour in the team's performance to reverse poor results. This endeavour might involve taking bolder risks to achieve positive outcomes, potentially necessitating a shift in strategy and alterations to the team's playing style in the closing matches before their eventual dismissal. Consequently, this approach shift could lead players to engage in higher-intensity activities.

What stands out from these values is that in the last 4 games of each coach, the intensity was higher when compared with the first 4 games (only the last HC in our sample doesn't have his last 4 games); meanwhile, in training, the values were almost equal for each coach status. With an increase in the game demands and with the need that training drills should be designed considering the magnitude of the intensity of the gameplay, for example, in MM-G (43), there should have been an increase in training intensity, which was not verified according to the average values obtained in the last 4 weeks of training of the dismissed HCs. Subsequently, players may not be prepared to have the best possible performance and run the risk of contracting injuries, conditioning the options of coaches in a likely contestation context and possible loss of employment.

Furthermore, beyond these interesting results, our study methodologically ensures a context of analysis that has not previously been explored. Previously, studies in this area were focused mainly on analyses aimed at understanding whether the dismissals of HCs, combined with the arrival of the new ones, would or would not improve the teams’ results. Very little research was related to the physiological effects and what influence they could have after the dismissals (44). Our study, using more than one HC during the research period in the same club context and by normalizing the number of games and training sessions to be considered, guaranteed a predominantly physiological context for analyses, adding different parameters to consider and complementing previous studies focused on sporting results.

## 5. Study limitations

An important factor that can constrain the analyses conducted is the composition of the training weeks under examination. While the weeks scrutinized were composed of open microcycles, featuring only one game on the schedule, the preceding weeks leading up to the final 4 weeks of analysis for each coach may have entailed one or two games per week. This variation in the game schedule may have influenced the distribution of loads, training volumes, and intensities in the subsequent weeks, owing to the routine fatigue management inherent in high-performance soccer. In addition, contextual factors were not considered for analysis. The quality of the opposition, the match status, the location or even the playing style are some contextual factors that can also affect the physical responses given by the players and thus may present a strong influence on the match's physical performance (40, 41).

## 6. Practical implications and future research

Understanding the process of replacement of an HC in professional soccer and how it can affect the players' performance can help to support changes in terms of tactical preparation and playing style, “mechanising” motor patterns and generating accurate motivational strategies to adopt during training and competition. Usually, the previous HC is fired after a negative streak of results, and the upcoming HC has to handle worst-case scenarios, escalating the pressure on him caused by the urgent need to win. Having this knowledge of how players probably can behave after a replacement of an HC can increase the chances of optimizing training load planning game preparation and avoid large variations that could negatively affect the players' fitness and create an environment conducive to generating even more stress than that caused by poor sports performance.

For future research, it is intended to (1) carry out a study of the dependence relations of locomotor intensity indicators between training and game contexts, checking whether there is an association between training intensity and performances obtained in the game, in and between coaches; (2) Analyse and compare the locomotor intensity indicators of the players between a coach replacement process and a continuity process.

## 7. Conclusions

The effects of HC replacement on the physiological component require careful analysis and further research. When comparing the effects of volume and intensity in external load metrics during competition, it's noticeable that players tended to achieve higher values under dismissed coaches. Conversely, during training, retained coaches generally had higher values across most external load metrics as well as the internal load metric (RPE-T). From these findings, it is possible to conjecture that when HC arrive, they may tend to change the dynamics and intensity of training, but when they are in the pre-dismissal period (last 4 weeks), that same training intensity decreases. As a result of these data, MM-G, covered individually by the players, stands as a metric with the potential to, in a referential way, through its average values, function as a physical marker to alert for performance decline that could lead to dismissal of an HC.

## Data Availability

The datasets presented in this article are not readily available because of legal policy. Requests to access the datasets should be directed to honoratosousa@hotmail.com.
